# Colorectal Cancer Stem Cells and Cell Death

**DOI:** 10.3390/cancers3021929

**Published:** 2011-04-11

**Authors:** Veronica Catalano, Miriam Gaggianesi, Valentina Spina, Flora Iovino, Francesco Dieli, Giorgio Stassi, Matilde Todaro

**Affiliations:** 1 Department of Surgical and Oncological Sciences, University of Palermo, Via Liborio Giuffrè 5, 90127 Palermo (PA), Italy; E-Mails: catalano1981@libero.it (V.C.); v.spina81@libero.it (V.S.); flora.iovino@gmail.com (F.I.); dieli@unipa.it (F.D.); matilde.todaro@gmail.com (M.T.); 2 Department of Cellular and Molecular Oncology, IRCCS Fondazione Salvatore Maugeri, Via Salvatore Maugeri, 27100 Pavia (PV), Italy; 3 Departement of Biopathology and Medicine Biotechnologies, University of Palermo, Via Liborio Giuffrè 5, 90127 Palermo (PA), Italy; E-Mail: dieli@unipa.it

**Keywords:** cancer stem cells, apoptosis, BMP4, survivin

## Abstract

Nowadays it is reported that, similarly to other solid tumors, colorectal cancer is sustained by a rare subset of cancer stem–like cells (CSCs), which survive conventional anticancer treatments, thanks to efficient mechanisms allowing escape from apoptosis, triggering tumor recurrence. To improve patient outcomes, conventional anticancer therapies have to be replaced with specific approaches targeting CSCs. In this review we provide strong support that BMP4 is an innovative therapeutic approach to prevent colon cancer growth increasing differentiation markers expression and apoptosis. Recent data suggest that in colorectal CSCs, protection from apoptosis is achieved by interleukin-4 (IL-4) autocrine production through upregulation of antiapoptotic mediators, including survivin. Consequently, IL-4 neutralization could deregulate survivin expression and localization inducing chemosensitivity of the colon CSCs pool.

## 1. The Cancer Stem Cells (CSCs) Hypothesis

During the past years the process of tumorigenesis was explained by cancer biologists through the stochastic model, according to which all tumor cells share common genetic and epigenetic mutations, reflective of their clonal origin [1]. In addition to the genomic instability, intrinsic factors (levels of transcription factors, signaling pathways) and extrinsic ones (host factors, microenvironment, immune response) influence tumor cells behavior leading to significant heterogeneity in terms of features, surface markers expression, proliferation kinetics and tumor initiation capacity [2].

**Figure 1.**
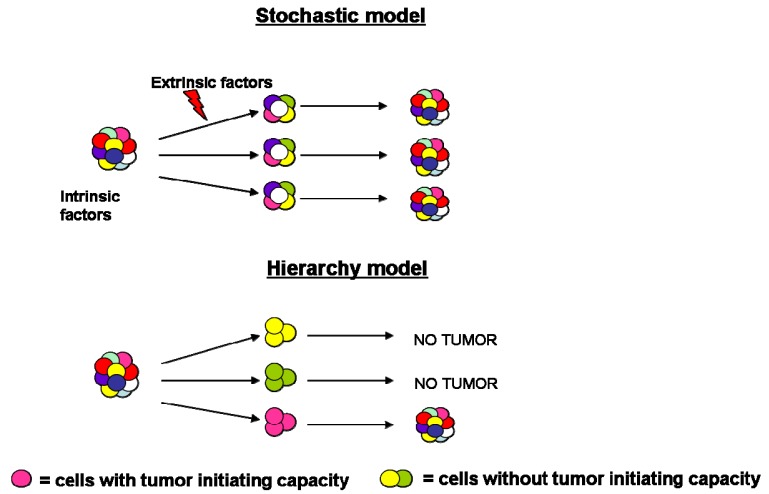
Models of tumor heterogeneity. Tumor heterogeneity has been explained by two theories: according to the stochastic model, tumor cells are influenced by intrinsic and extrinsic factors; by contrast, in the hierarchy model, tumor cells have different functional abilities and only a subset can initiate tumor growth.

More recently, the hierarchy model has been proposed, according to which cancer consists of a heterogeneous population characterized by various stages of differentiation. Accumulating evidence has posited that tumor mass is characterized by the presence of a small population of cells, necessary and sufficient to initiate and sustain indefinitely tumor growth and subsequent progression. These “tumor-initiating cells” are also called cancer stem cells (CSCs) since they share the hallmarks of normal stem cells (e.g., unlimited self-renewal, quiescence, multipotentiality and expression of drug and apoptosis resistance genes), expand the stem cell compartment undergoing symmetric division and differentiate into the multiple lineage via asymmetric division [3] (Figure 1).

Dick and colleagues demonstrated that only a small minority of acute myeloid leukemia (AML) cells were able to produce leukemia in NOD/SCID murine model [3].

From the initial studies in hematological malignancies, CSCs have been identified in a variety of solid tumors including breast, prostate, brain colon, pancreas, ovary, lung, and, recently in thyroid, as assayed either by their *in vitro* clonogenity or by their ability to initiate new tumor growth after xenotransplantation into immunocompromised mice which recapitulate the phenotypic heterogeneity of the primary tumor [4-11].

The emergence of CSCs and subsequent cancer development may arise from deregulation of the processes that regulate self-renewal, cell fate and differentiation of normal stem or progenitor cells [12], but moreover CSCs may originate from mutations in differentiated cells favoring timeless proliferative potential [13].

Several signaling pathways such as Wnt, Notch and Sonic Hedgehog (Shh) have been found to regulate the self-renewal of normal stem cells in a variety of cancers.

The importance of a self-renewal pathway in maintaining Leukemia Stem cells (LSCs) has been first underlined by Jamieson group. Their results showed that an aberrant activation of Wnt pathway is implicated in human blast crisis LSCs propagation. They also identified an increased activation of Wnt signaling in breast CSCs growth. Shh signaling pathway is also known to play a critical role in maintaining human LSCs, breast, glioblastoma and colon CSCs. Finally, Notch pathway has been shown to be activated in colon CSCs subset but also in breast and glioblastoma CSCs [14].

## 2. Colorectal Cancer Stem Cells

Normal colonic stem cells (NCSCs) are localized at the base of the crypts surrounded by intestinal subepithelial myofibroblasts (ISEMFs). Defined by properties of self-renewal and multilineage differentiation, they ensure a high rate of tissue renewal: by asymmetric division NCSCs generate another SC and a progenitor cell also known as a transit-amplifying cell (TAC) which, in turn, generates more mature cells of colonic epithelium. It has been suggested that ISEMFs play a critical role in the regulation of a correct balance between SCs self-renewal and differentiation, by paracrine secretion of growth factors and cytokines [13,15].

In addition to ISEMFs, maintenance of colonic epithelial SCs niche is modulated by high Wnt activity in the lower region of the crypt which induces the expression of EphB receptors and the subsequent interaction with ephrin ligands located in the higher position of the crypt [13,16].

Another signaling pathway identified as a key regulator of the SCs niche is that mediated by Bone Morphogenetic Proteins (BMPs). As a consequence of the high expression of BMP antagonists in the colon bottom, the BMP activity is higher in the upper region of the crypt inducing differentiation of colonic epithelial cells [13,15].

In 1990, Fearon and Volgestein suggested a genetic model for colorectal tumorigenesis in which gene mutations occurred with a specific time defining a particular stage of tumor development [17].

In patients with familial adenomatous polyposis, mutations in the *Adenomatous Polyposis Coli* (APC) gene are reported as the initiating gatekeeper regulating positively Wnt machinery and causing hyperproliferation and early adenoma formation [18].

The stage of intermediate adenoma is promoted by B-RAF and K-RAS mutations. Late adenoma results from loss of heterozygosis involving the chromosome 18q, mutations in *Small Mother against DPP homolog 4 (Smad4), Cell Division Cycle 4 (CDC4)* and *Delected in Colorectal Cancer* (DCC) or alternatively mismatch repair deficiency. P53, Bax and insulin-like growth factor receptor2 mutations are responsible for invasive cancer; lastly, unknown factors lead to metastatic cancer [13,19].

Even in cancers caused by alterations in genomic integrity, neoplastic change might initiate through subsequent mutations in morphogenetic pathways regulating normal proliferation of intestinal epithelium, such as Akt/ PKB, Wnt, Shh, Notch and BMPs [18]. These multiple genetic mutations, restricted to TACs, would be acquired by their progeny resulting in increased proliferative potential, independence of extrinsic growth control signals and autonomous control over all metabolic activities that feed tumor progression [20].

Although it has long been assumed that neoplastic formation derives from alterations within adult colonic stem cells, the existence of colorectal cancer stem cells (CR-CSCs) has been demonstrated through the finding that colon CD133^+^ cells are able to grow exponentially *in vitro* as undifferentiated tumor spheres, when cultivated in serum-free medium, and initiate tumor growth in mouse models, thus reproducing the same morphological and antigenic pattern of the original human tumor [21-23].

Significant controversies have been raised about the use of CD133 as a CR-CSCs marker since Shmelkov *et al.* [24] showed in reporter studies of CD133 promoter that CD133 expression in epithelial tissues is not restricted to stem or progenitor cells but it is distributed among the differentiated luminal and ductal epithelial cells. In metastatic adenocarcinoma, the expression of CD133 was down-regulated and CD133^−^ subset formed more aggressive tumors than the same generated by CD133^+^ cells. Cumulatively, these data suggest that CD133 by itself does not exert a critical role in supporting tumor growth, thus confirming the need to identify biomarkers for CR-CSCs.

In this regard, Barker *et al.* [25] focus on Lgr5 protein (Leucine-rich-repeat-containing G-protein-coupled Receptor 5), highly expressed by murine crypt base columnar cells, defined as adult stem cells based on their ability to generate all epithelial lineages.

In addition, previous work of Stassi's group has provided evidence that mammalian RNA-binding protein Musashi-1 (Msi-1) could be a potential stem cells marker, since it is abundantly expressed in CR-CSCs [23].

Many studies have provided proof that, within the CD133^+^ subpopulation, there exists a minority of cells possessing tumor-initiating ability.

Dalerba *et al.* [7] suggest cell surface glycoprotein CD44 and Epithelial Cell Adhesion Molecule (EpCAM) as specific markers of CR-CSCs: in the context of CD133^+^ tumor population, they have identified a subset of stem-like CD44^+^/EpCAM^high^ cells able to generate tumor xenografts upon serial transplantation into NOD/SCID mice. A further isolation of colon cancer cells using the mesenchymal stem cell marker CD166 enhanced the success of tumor xenograft. A recent study performed by Huang *et al.* [26] showed that enzymatic activity of ALDH1 can be used as a potential CR-CSCs marker being expressed by cells positive for CD44^+^ or CD133^+^ located at the base of normal crypts. It has been reported by the same group that during tumor progression the selection of CD44^+^, CD133^+^ cells with ALDH activity increases in number and reaches the crypt axis.

## 3. Apoptosis and CSCs Strategies to Evade Death Signals

An effective therapeutic approach against cancers would eliminate the pool of CSCs which are quiescent or slowly replicating and thus more resistant to apoptosis induced by current cytotoxic regiments [27].

**Figure 2.**
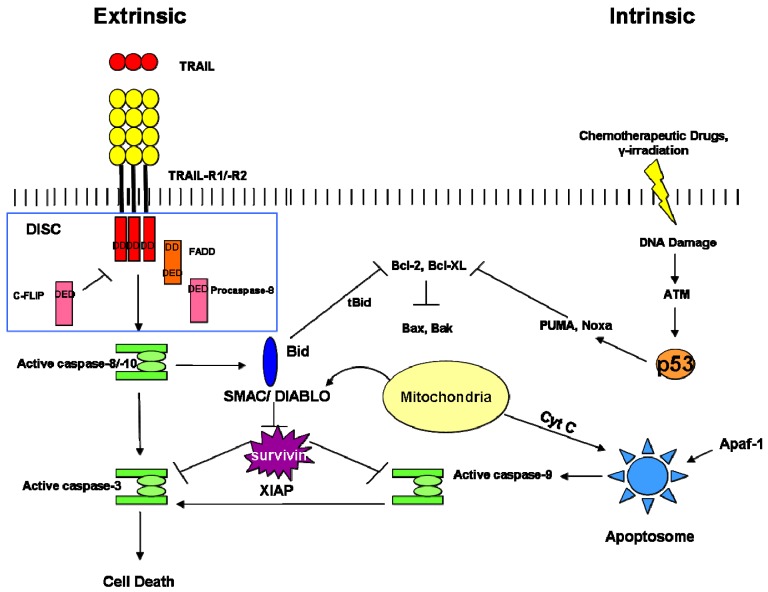
The two major processes that control apoptosis.

In the following section, we describe the strategies of death escape in CSCs, including NF-kB signaling, Bcl-2 family proteins over-expression, enhanced Inhibitor of Apoptosis Proteins (IAPs), increase of survival signals (survivin, cFLIP), which are correlated to the two major signaling pathways by which cells undergo apoptosis (Figure 2).

As its name implies, the intrinsic apoptosis pathway is triggered by cellular stress such as hypoxia, DNA damage or growth factor deprivation. p53-Mediated activation of the pro-apoptotic BCL2 family proteins, including PUMA, NOXA, BAX and BAK, induces mitochondrial outer membrane permeabilization (MOMP) and subsequent release from the mitochondria of apoptogenic factors such as cytochrome *c*. Once released, it binds APAF1 to form a complex, known as the “apoptosome”, which recruits pro-caspase 9 promoting its autocatalytic activation. Then, caspase 9 activates downstream caspases, including caspase 3, 6, and 7, leading to apoptosis [28].

The extrinsic apoptosis pathway is initiated upon ligand binding to the extracellular component of death receptors (DRs) of the tumor necrosis factor (TNF) superfamily, such as TRAIL-R (TNF-Related Apoptosis Inducing Ligand), CD95 and NGFR (Nerve Growth Factor Receptor). Multimerizated receptor molecules cause the recruitment of the Death Domain (DD) containing adapter molecule FADD (Fas-Associated Death Domain) which, in turn, via its second functional DD, recruits procaspases 8 and 10 to form DISC (Death-Inducing Signaling Complex) [29]. Self-cleaved, pro-caspase 8 and 10 activate effector caspases 3, 6, and 7, so triggering apoptosis.

There is a cross-talk between the extrinsic and intrinsic pathways since caspase 8 cleaves the pro-apoptotic BCL2 family member BID, which in turn engages the mitochondria through BAX and BAK, leading to activation of caspase 9 and further of caspases 3, 6, and 7 [30].

Escape from apoptosis is one of the hallmarks of any tumor-initiating cell.

CSCs survival has been linked to an increased expression of Inhibitor of Apoptosis Proteins (IAPs), a family of endogenous caspase inhibitors that inhibit the extrinsic and intrinsic pathways by binding to caspases and preventing their activity. Interestingly, higher levels of survivin, a member of IAP, have been observed in CD34^+^ fraction within hematopoietic stem cells compared to the CD133^-^ population [31].

Other evidence for the CSCs resistance to apoptosis induced by anticancer therapies comes from the observation that CD133^+^ glioblastoma cells showed a lower rate of apoptosis induced by radiation than the CD133^-^ differentiated fraction due to a strong activation of DNA damage checkpoint kinases Chk1 and Chk2 [32].

Another important negative regulator of death receptor-induced apoptosis is cellular FLICE-like inhibitory protein (cFLIP) that counteracts DR-induced apoptosis in CSCs via its ability to block signal transduction from activated death receptors by preventing the recruitment and activation of caspase-8 at DISC complex. Recently, high levels of cFLIP have been associated with the CD133^+^ fraction of glioblastoma SCs, compared with the CD133^-^ population [33].

Moreover, the transcription factor NF-kB has been connected to apoptosis inhibition by activating the transcription of many antiapoptotic genes. This notion has been confirmed in breast cancer where NF-kB inhibition by a small molecule inhibitor induces preferentially apoptosis in the CSCs and progenitors population while failing to eliminate normal SCs.

Among the various mechanisms of defective apoptotic signaling, the over-expression of anti-apoptotic proteins, including Bcl-2 and Bc1-XL, constitutes an invariable finding in CSCs.

In glioma SCs, high levels of Bcl-2 and Bcl-XL have been detected in the CD133^+^ subpopulation compared with the CD133^-^ fraction. Furthermore, glioma SCs resistance to treatment with a small-molecule inhibitor targeting to anti-apoptotic Bcl-2 family proteins, named ABT-737, was determined by high expression of the anti-apoptotic Bcl-2 protein, Mcl-1 [34].

Along similar lines, Ricci-Vitiani and colleagues discovered that in primitive neural cells an innate resistance to apoptosis, induced by death receptors, was due to the absence of caspase-8 and elevated levels of antiapoptotic protein PED/PEA-15 [35].

There is emerging evidence that many aspects of the extrinsic pathway are also dysregulated in CSCs. Stimulation of TRAIL receptors has potential application as anticancer therapy because they induce apoptosis preferentially in tumor cells, also in those with p53-inactivating mutations, sparing normal cells. It has been shown that in p53-deficient colon cancer cells Apo2L/TRAIL induces apoptosis cooperating with chemotherapy. In particular, two classes of pro-apoptotic receptor agonists (PARAs) have been developed to trigger activation of the extrinsic apoptotic pathway: recombinant human Apo2L/ TRAIL and monoclonal agonist antibodies [36].

Nevertheless, many tumor cells are resistant to TRAIL such as the stem cell-like CD133^+^ glioma cells in which this phenomenon could be correlated to down-regulation of caspase-8 induced by promoter methylation [37].

For this reason, it has been proposed to combine administration of TRAIL with chemotherapy. First results have been obtained *in vitro* with acute myeloid progenitors because their growth has been significantly reduced by recombinant TRAIL alone and in combination with Ara-c and daunorubicin [34].

Stassi's group has recently provided evidence that microenvironmental niche contributes to the refractoriness of CSCs by facilitating proliferative effects over apoptotic death signals. Several *in vitro* and *in vivo* experimental data showed that colon CSCs are protected from cell death by autocrine production of IL-4 which promotes cell survival and expansion by stimulating the expression of antiapoptotic genes, including cFLIP, PED and Bcl-xL. Therefore, the administration of anti-IL-4 neutralizing antibody or IL-4 receptor α antagonist increases sensitivity to death receptor- and cytotoxic drug-induced cell death of CR-CSCs.

With this in mind, additional investigations are needed to evaluate whether the autocrine survival signal of IL-4 might represent a link between cell survival and stemness in order to direct therapeutic strategies [38].

## 4. Survivin

Survivin is the smallest member of the IAP family, also known as API4 (Apoptosis inhibitor 4) or BIRC5 (baculoviral IAP repeat-containing protein-5).

Structurally, survivin is characterized by a single baculovirus IAP repeat (BIR domain) in the N-terminus region that stretches from amino acid residue 15 to 87. This domain is crucial for dimer formation and other protein-protein interactions, such as with effector caspases [39,40]. The carboxyl-terminal end lacks RING zing finger (Really Interesting New Gene) and CARD (Caspase Recruitment Domain) domains.

Survivin includes three introns and four exons and alternative splicing of its pre-mRNA gives rise to four transcripts, which encode different proteins: survivin full length (SFL), survivin 2B, survivin ΔEx3, survivin 3B and survivin 2α [41].

SFL is a 142 amino acid protein derived from exon 1-4; Survivin 2B in addition to 1-4 exons, presents a little portion derived from intron 2. SurvivinΔEx3 is generated from exons 1, 2 and 4 with exon 3 skipped. The isoform Survivin 3B, recently reported, arises from insertion of exon (3B), that results in a stop codon with production of a 120 amino acid protein. Survivin 2α includes only exons 1 and 2, resulting in a truncated protein of 74 amino acids.

Both SFL and survivin ΔEx3 carry anti-apoptotic properties, whereas survivin 2B plays a proapoptotic role and sensitizes resistant leukemia cells to chemotherapy in a p53 dependent manner [42]. Survivin 2α attenuates the anti-apoptotic activity of SFL by physical interaction, as suggested by immunoprecipitation assay. Survivin 3B is an anti-apoptotic isoform, but it is not involved in cell cycle regulation [43].

SFL plays a key role in two essential cellular processes: inhibition of apoptosis and promotion of cell proliferation [44,45]. Two fundamental conditions appear to be essential for its anti-apoptotic role: the homodimerization and the phosphorylation on residue Thr 34 [46,47], promoted by cdc2 kinase that colocalizes with survivin in mitotic machinery [48] of dividing cells. Survivin binds centromere of metaphase chromosomes, helping chromatid segregation [49,50] and microtubules stabilization in late apoptosis [51].

Survivin is usually expressed in embryonic tissues and decreases in normal cells, but the majority of tumors, with poor prognosis, present up-regulation of the protein.

Nowadays, the mechanism by which survivin regulates apoptosis remains unclear; according to the old idea, survivin, as other mammalian IAPs, acts directly or indirectly as an endogenous inhibitor of caspases. Survivin was found to interfere with both extrinsic and intrinsic factors (Fas stimulation, TRAIL, over-expression BAX, caspase 3, caspsase 7 and caspase 8) [52-55]. It could be found in a convergence point of the two apoptotic pathways at the level of final effectors: caspases 3 and 7, inhibiting their activation. Other experiments suggest that survivin inhibits only active caspase 9 by its association with X-linked IAP [56].

A further mechanism by which survivin may inhibit apoptosis is by acting on the tumor suppressor p53, through caspase 3/ Mdm2 axis [57]. P53 is able to induce a block of cell cycle progression and/or apoptosis. Mdm2 is a negative regulator of p53 (inhibiting its expression and activity) [58] and a caspase substrate [59], since it is cleaved by caspase 3 during p53-mediated apoptosis.

The survivin promoter has a p53 binding element and it is reasonable to think that p53 induces apoptosis antagonizing the anti-apoptotic activity of survivin [60]. Its involvement in apoptosis and proliferation, crucial processes for cancer growth and progression, makes survivin an attractive target for anti-cancer therapy. APC gene mutations, a key initial mechanism in colon carcinogenesis, could be responsible for this over-expression [61]. In normal conditions, APC should suppress survivin expression in the crypt's middle region, where SCs should be induced to apoptotic death on the top of the crypt. In accordance with this hypothesis, the bottom of the normal crypt contains SCs and high levels of survivin that would block apoptosis and contribute to prolonged survival of these cells. In the middle region of the crypt, survivin expression decreases and leads to cell proliferation block, with subsequent differentiation and maturation. Instead in apical portion, survivin levels are nearly absent, because cells undergo differentiation and then apoptosis. On the contrary, in APC mutant crypts, survivin expression increases blocking apoptosis and contributing to cell immortality.

Interestingly, Wnt/β-catenin and Notch pathways control both normal SCs expansion [62,63] and survivin expression [64]. So, deregulation of these pathways in CSCs induces upregulation of survivin, contributing probably to carcinogenesis.

For all these reasons, it could be advantageous to find a strategy that affects survivin function and expression for selective targeting SCs.

For example, IL-4 neutralization has been proposed as an innovative approach to decrease survivin levels. IL4 binding to its receptor (IL-4Rα) actives two related pathways: JAK/STAT, which leads to downstream phosphorylation and activation of STAT6 transcriptional factor, and IRS-2 (Insulin Response Substrate) signaling with consequential activation of downstream kinase Akt, crucial for cell proliferation and apoptosis resistance [65].

Recently, a possible link has been shown between IL-4 pathway and survivin expression in CR-CSCs [66]. In fact CR-CSCs express survivin and its levels decrease after IL-4 neutralization. Interestingly, IL-4/STAT-6 signaling pathway inhibition affects both survivin expression and localization, increasing its nuclear pool that allows apoptosis and correlates with better prognosis in a range of malignant tumors.

Another strategy for targeting survivin involves anti-sense oligonucleotides (AO) and small interfering RNA (siRNA), preventing protein translation: survivin siRNA induces caspase-independent apoptosis [67]. Moreover, imidazolium-based compound YM155, currently in phase II clinical trials, is a small molecule that binds and blocks the activity of the survivin promoter, inhibiting in this way its gene transcription.

Unlikely, survivin inhibitors have moderate antitumor effect in clinical trials and it could be useful to test their combination with conventional treatment.

## 5. Clinical Implications of CSCs

The discovery of CSCs in a variety of tumors has changed the view of carcinogenesis and therapeutic strategies. According to the stochastic model, the tumor chemoresistance is due to pre-existing clones with mutations that confer drug-resistance. The CSCs model postulates that CSCs evade death signals induced by current therapeutic drugs through a variety of strategies including up-regulation of multidrug-efflux pumps able to exclude exogenous substances, alterations in DNA-repair mechanisms, altered cell cycle checkpoint controls and impaired apoptosis machinery. In addition, CSCs survive to current treatments, evaluated for the ability to kill only more differentiated and highly proliferating cells, because CSCs are proliferatively quiescent, less differentiated and overcome apoptosis resistance evading the control mechanisms.

A combination of 5-FU, oxaliplatin and leucovorin (referred to as FOLFOX) and a combination of 5-FU, oxaliplatin and irinotecan (referred to as FOLFIRI) are the current therapy for colon cancer patients. Actually, the therapeutic approach for CRC includes anti-VEGF or EGFR monoclonal antibodies which improve positive outcomes in patients suffering from metastatic colon cancer and severe hepatic dysfunction [13]. However, none of these anticancer therapies is curative in most patients with metastatic disease due to failure to eradicate the CSCs compartment.

**Figure 3.**
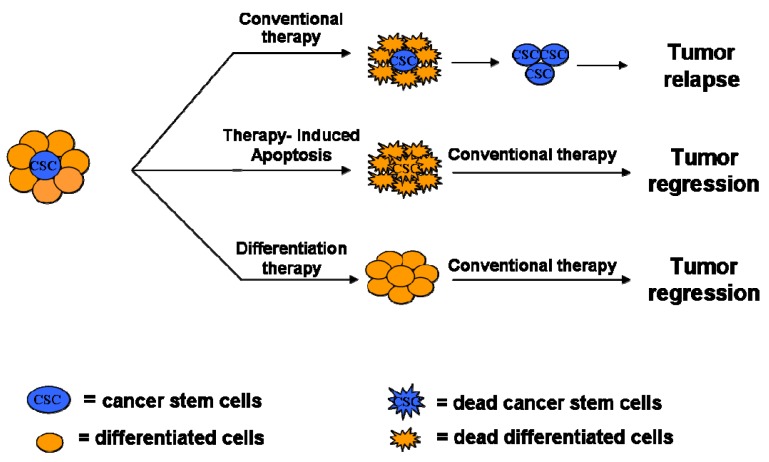
Therapeutic Strategies for CSCs sensitization.

The development of targeted therapies for this cancer type would therefore require a better knowledge of the different aspects of stem cell biology in the context of CRC such as complex network of mechanisms that regulate tumor development and resistance to chemotherapy. It is therefore evident that a therapeutic approach to selectively target CSC pool bypassing their chemoresistance could be more effective to eradicate bulk tumor. Thus, the purpose of new therapeutic regiments is to eliminate the self-renewal compartment of tumor mass by:

- targeting stem cell properties inducing the inactivation of survival pathways in CSCs.
- forcing CSCs to differentiate [1] (Figure 3).

## 6. BMPs: An Example of Differentiation Therapy

Considerin the role of Bone Morphogenetic proteins (BMPs) in development and differentiation stages, these molecules have been studied over the past decade in tumorigenesis and metastasis formation.

BMPs belong to a subgroup of the transforming growth factor-beta (TGF-β) super-family; so far 20 BMPs have been discovered [68]. According to current models, BMPs bind two distinct serine/threonine kinase receptors; different combination of type I and type II receptors determine the specificity for the ligands. Upon ligand binding, the type II receptor *trans*-phosphorylates type I receptor in its GS domain; initiating the signal transduction by phosphorylating Smad1/5/8 proteins (RSmads). Then RSmads form a complex with Smad4 (CoSmad) and translocate into the nucleus, where this complex could bind directly to gene regulatory elements or interact with other transcription factors regulating target gene expression [69]. In addition to the Smad pathway, BMPs activate an alternative pathway, which includes p38 and ERK MAP kinases [70]. Moreover, BMPs activation is tightly regulated by the presence or the absence of antagonists, such as Gremlin, Chordin and Noggin [71].

Originally these proteins have been studied and characterized for their chondrogenic and osteogenic abilities, as they are able to induce ectopically bone formation in rodents [72]. Afterwards, BMPs were analyzed for their role in cell growth, differentiation and apoptosis.

It is well established that several BMPs have a function in multiple developmental processes. Studies in *Drosophila melanogaster* and *Xenopus laevis* established that BMPs are required for correct dorsal-ventral axis formation and mesoderm induction in embryos [73,74]. Since these data prove that BMPs pathway is essential for the development of embryos invertebrates, further studies were carried out on murine models to strengthen the hypothesis that BMPs are important during vertebrate embryogenesis. Many knockout mice were generated for BMPs, BMPs receptors and molecules involved in the signaling pathway. Most of these models (BMP2, BMP4, BMPR I and II, Smad 4 and 5 KO mice) are lethal, as mutant embryos, exhibiting multiple gastrulation defects, among which include lack of mesoderm formation and incorrect left-right axis asymmetry, morphogenesis and organs positioning [75-80]. A different phenotype was observed in BMP7 null mice: these mice present postnatal lethal mutations with various developmental skeleton-kidney and eye defects. Given that BMPs have a role in embryonic development, it was supposed that these proteins may play a role during SCs differentiation. Pera *et al.* [81] demonstrate that human embryonic SCs treatments with Noggin impair their spontaneous differentiation, suggesting that in these cells BMPs pathway activation induces differentiation.

As mentioned before, in the colon crypts ISEMF cells contribute to stem cells niche maintenance balancing different and opposite signals that promote self-renewal (Wnt and Notch pathways) and differentiation (BMPs pathway). The understanding of these mechanisms is important because it is hypothesized that the existence of a CSCs niche may have a role in maintaining and increasing the CSCs pool [82]. In CRCs, an abnormal activation of Wnt signaling pathway leads to nuclear β-catenin accumulation and subsequent abnormal CSCs proliferation; moreover, BMPs signaling inhibition promotes nuclear β-catenin activity through PTEN inactivation and activation of PI3K-Akt pathway [83]. A subsequent microarray study identifies a list of genes differentially expressed in colon bottom crypts and in the tops: the first group includes genes involved in Wnt and Notch pathways, but also BMPs inhibitors, such as Gremlin 1, Gremlin2 and Chordin-like 1; the second genes involved in BMPs and apoptosis pathway and cell cycle inhibitors [15]. These data suggest that in the colon bottom crypts a balance between Wnt/Notch and BMPs pathways is necessary in order to maintain and regulate CSCs niches.

More evidence for their putative role in CRC was provided by genetic studies and transgenic mice models. Germline mutations in genes encoding SMAD4, BMPRIA and BMP4 are found in up to 50% of individuals with juvenile polyposis, an autosomal dominant syndrome with a high risk for CRC [84-86]. Furthermore, Noggin transgenic mice phenocopy the intestinal histopathology of patients with this syndrome [87]; subsequently it was described that mice with an inducible mutation of BMPRIa develop intestinal polyps [83]. This body of data argues that BMPs signaling disruption leads to pre-cancerous lesions [88].

CRC develops as a result of increasing proliferation and apoptosis deregulation and TGF-β signaling inactivation have a key role in this pathology [89]. It has been reported that SMAD4 is frequently deleted in CRC and that BMPs pathway is inactivated in the majority of colorectal tumors [90,91]. Indeed, BMP2, BMP3, BMP4 and BMP7 inhibit proliferation and induce apoptosis and differentiation in colon cancer cells that do not have Smad4 mutation and loss of PTEN [88,92-94].

Considering BMPs' role in regulating SCs differentiation and inducing apoptosis and differentiation, it is possible to suppose that CSCs treatment with these molecules could induce differentiation and following chemotherapies sensitization. Some preliminary studies have been performed on both CSCs of glioblastoma and CRCs. Preliminary studies in glioblastoma demonstrate that BMP2, BMP4 and BMP7 treatment inhibits sphere forming and induces differentiation of CD133^+^ cells; moreover CD133^+^ cells pre-treatment with these cytokines attenuates tumor formation in mice [95-97]. Recently, the same results are obtained in CR-CSCs. The treatment of CD133^+^ CR-CSCs with BMP4 induces *in vitro* differentiation and reduces their tumorigenic potential; moreover *in vivo* the combined treatment with BMP4 and conventional chemotherapeutics reduces the tumor size [98]. These data open the possibility to use BMPs or analogue-drug to induce the differentiation of CSCs and to make them more sensitive to conventional chemotherapy.

## 7. Concluding Remarks

CSCs are believed to play a critical role in tumor initiation and recurrence. Current chemotherapeutic regiments target the most actively cycling cells, which represent the tumor bulk, sparing the CSC compartment. Thus, novel and more efficient stem cell-based therapies, able to kill this chemotherapy-refractory population, are needed to improve patients' survival. In this scope, the identification of agents that can inhibit the CSCs survival machinery forcing apoptosis or induce their differentiation represents the first step to achieve in the near future, providing important advances for cancer treatment.
